# Polymorphisms of *CHRNA3* and *CHRNA5*: Head and neck cancer and cigarette consumption intensity in a Brazilian population

**DOI:** 10.1002/mgg3.998

**Published:** 2019-10-09

**Authors:** Mariana R. Silva, Gilka J. F. Gattás, Juliana De Antonio, Isabela Firigato, Otávio A. Curioni, Fernanda de Toledo Gonçalves

**Affiliations:** ^1^ Departamento de Medicina Legal e Ética Médica Medicina Social e do Trabalho Instituto Oscar Freire LIM‐40 Faculdade de Medicina FMUSP Universidade de São Paulo São Paulo Brazil; ^2^ Departamento de Cirurgia de Cabeça e Pescoço e Otorrinolaringologia Hospital Heliópolis São Paulo Brazil

**Keywords:** *CHRNA3*, *CHRNA5*, cigarette consumption, head and neck cancer

## Abstract

**Background:**

Cigarette consumption has been identified as the main non‐etiological factor in head and neck cancer (HNC) development. One of the main compounds in cigarettes is nicotine, which binds directly to nicotine acetylcholine receptors (nAchRs) in the body, which are encoded by different genes of the *CHRNA* family. Polymorphisms in some of these genes have been studied in relation to the risk of HNC and cigarette consumption intensity. The aim of this study was to evaluate whether there were associations between the *CHRNA3* (rs578776) and *CHRNA5* (rs16969968) polymorphisms and HNC risk and between the polymorphisms and the intensity of cigarette consumption.

**Methods:**

A total of 1,067 individuals from Heliopolis Hospital in São Paulo were investigated, including 619 patients with HNC and 448 patients without diagnosed tumors. All participants answered a questionnaire about sociodemographic information and cigarette consumption data. The polymorphisms were determined by TaqMan genotyping by real‐time PCR.

**Results:**

The polymorphisms studied, rs578776 (*CHRNA3*) and rs16969968 (*CHRNA5*), did not have an association with HNC risk, but the rs16969968 homozygous genotype was associated with increased cigarette consumption intensity (OR 1.93, 95% CI 1.05–3.58).

**Conclusion:**

The polymorphism *CHRNA5* can be considered an indirect risk factor for neoplasms in these Brazilian samples when cigarette consumption increased.

## Graphical abstract

The gene *CHRNA5*, that encoded the α5 subunit of nicotinic receptor, increased the cigarette consumption intensity in Brazilian sample. Once tobacco consumed is a risk factor to the head and neck cancer development, these polymorphism can be considered an indirect risk factor for these neoplasm.

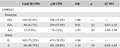

## 1 INTRODUCTION

Tobacco consumption is one of the most common public health problems worldwide, and it is widely consumed via cigarettes (Wen, Jiang, Yuan, Cui, & Li, 2014). Tobacco has been identified as the main risk factor for head and neck cancer (HNC) development (Colombo & Rahal, 2009; Lacko et al., 2014). HNC is a disease that includes tumors in the regions of the upper aerodigestive tract, such as the oral cavity, larynx, and pharynx. HNC represents the *sixth most common cancer worldwide* and affects more males than females, around 50 years, and with low income (Colombo & Rahal, 2009; Döbrossy, 2005; Simard, Torre, & Jemal, 2014). In Brazil, in 2018 and 2019, there were approximately 14,700 new cases of oral cancer and 7,670 cases of laryngeal tumors (Instituto Nacional de Câncer José Gomes da Silva, 2018).

There are more than 4,000 different substances in cigarettes, and nicotine is the main ingredient related to their dependence (Siqueira, 2017). After being inhaled, the nicotine can be easily absorbed in the alveoli and enter the systemic circulation, where it is then transported quickly to the brain. This short time period leads to reinforced use, resulting in neuroadaptation (Siqueira, 2017).

Nicotine acts directly in our body by binding to nicotine acetylcholine receptors (nAchRs), which are distributed in the central and peripheral nervous system (Changeux, 2010) and muscle, endothelial, and immune cells (Siqueira, 2017). These receptors are ligand‐gated ion channels composed of five subunits, which can be formed by 12 different isoforms, nine α subunits, and three β subunits (Bierut et al., 2008; Siqueira, 2017). The receptor responds to acetylcholine, nicotine, and agonists, and the channels are permeable to cations (Changeux, 2010); some presynaptically located receptors can modulate the release of certain types of neurotransmitters, for example, dopamine, which is a neurotransmitter linked to the reward reinforcement system and pleasure (Herman, DeVito, Jensen, & Sofuoglu, 2014; Wen et al., 2014). The arrangement between these 12 subunits results in receptors that differ in distribution throughout the body, pharmacological properties, and nicotine stimulation responses (Siqueira, 2017; Wen et al., 2014). Repeated exposure to nicotine causes a decrease in the nAchRs response, leading to a desensitization of these receptors (Changeux, 2010). Therefore, an increase in nicotine consumption is needed to result in the same response linked to dopamine effects (Siqueira, 2017; Van Skike et al., 2016).

Nicotine receptor subunits are encoded by *CHRN* which locate on chromosome 15q25.1, the *CHRNA5‐A3‐B4* cluster encoding α5, α3 and β4 subunits, respectively, which may have polymorphisms that have been studied in relation to cigarette smoking and some kinds of diseases. Two variants in these regions, rs578776 (*CHRNA3,* OMIM:118503) and rs16969968 (*CHRNA5*, OMIM:118505), have been associated with both smoking behavior, including smoking quantity, and certain types of neoplasms, for example, HNC (Anantharaman et al., 2014; Changeux, 2010; Lips et al., 2010). The associations of these polymorphisms have also been studied in lung cancer; some of these studies found that the variations are related to smoking quantity and that cigarette smoking is a risk factor for this type of neoplasm. The authors suggest that the polymorphisms indirectly influence the risk of lung cancer (Thorgeirsson et al., 2008). However, other studies have not found an association between smoking behavior and these polymorphisms and suggest that variants influence the risk of lung cancer directly (Amos et al., 2008; Hung et al., 2008). Thus, there is still no consensus on how these polymorphisms are related to this type of cancer, whether directly or indirectly.

Since the two polymorphisms have been associated with cigarette consumption, this is one of the risk factors for HNC, and the function of these variants in the *CHRNA*5/A3/B4 gene cluster in this neoplasm has not been intensively investigated. The aim of this study is to investigate a possible association between the *CHRNA3* (rs578776) and *CHRNA5* (rs16969968) variants and the risk of HNC and the intensity of cigarette consumption in a Brazilian sample population.

## 2 METHODS

### 2.1 Ethical compliance

The Ethics Committee for Research Projects Analysis of the University of São Paulo, School of Medicine and the Ethics Committee of Heliopolis Hospital of São Paulo, Brazil approved the present study.

### 2.2 Population

In this study, we evaluated 1,067 individuals, both gender, over 18 years old, which included 619 patients with a histologically confirmed HNC diagnosis, who were recruited during 2000–2011 from the Heliopolis Hospital. From the same hospital, the control group was recruited including 448 patients with no tumor diagnosis and familial HNC history, frequency matched with HNC patients for gender and age within 5 years. The control group included only individuals recently diagnosed with diseases unrelated to tobacco or alcohol, such as digestive system disorders, injuries and diseases of the musculoskeletal system and connective tissue, genitourinary system, or circulatory system. All participants answered a standardized questionnaire on age, gender, skin color, education, and smoking status (nonsmoker, former smoker, or current smoker). Subjects who had stopped smoking for at least 12 months before the interview were classified as former smokers, and those who still smoked until the interview were classified as current smokers. Participants signed an informed consent form approved by the Ethics Committee. The minimum frequency necessary to obtain a significant sample size was 358 cases and 358 controls.

### 2.3 DNA extraction

Genomic DNA was assessed from 5 ml of peripheral blood lymphocytes through the salting‐out extraction method (Miller, Dykes, & Polesky, 1988), wherein the whole volume of blood was treated twice with erythrocyte lysis buffer (1‐mM NH4HCO3; 114‐mM NH4Cl) for 15 min each time. Next, lymphocytes were incubated overnight at 37°C with 0.01 ml of TEN buffer (0.1‐M Tris‐Cl; 0.01‐M EDTA; 1‐M NaCl), 0.2 ml of 10% sodium dodecyl sulfate (SDS), and 0.5 ml of a proteinase K solution. Saturated NaCl solution (6 M) was then added and mixed. The pellet was discarded, and the supernatant was washed with absolute ethanol to precipitate the DNA, which was eluted in TE solution (buffer solution with Tris‐ HCL + EDTA [Ethylenediamine tetraacetic acid]) (10‐mM Tris; 1‐mM EDTA). After extraction, the genomic DNA quantity and quality were assessed under wavelengths of 260 nm (A260) and 280 nm (A280) using a spectrophotometer (NanoDrop 2000, Thermo Fisher Scientific™). An A260/A280 ratio between 1.8 and 2.2 was used to classify the samples as high genomic DNA quality.

### 2.4 Genotyping and statistical analysis

The *CHRNA*3 (rs578776‐NG_016143.1) and *CHRNA5* (rs16969968‐NG_023328.1) polymorphisms were determined using TaqMan single nucleotide polymorphism (SNP) Genotyping Assays C_721253_10 and C_26000428_20, respectively, whose amplifications were conducted according to the manufacturer's instructions and performed on a Step One‐Plus Real‐Time PCR System (Applied Biosystems).

The Hardy–Weinberg equilibrium was evaluated using the *χ^2^* test. To assess the possible association between polymorphisms and HNC risk, we used logistic regression analysis, which considered HNC as a dependent variable and the polymorphism, gender, skin color, age, and education level and tobacco consumption as covariates in the model. Odds ratios (ORs) with 95% confidence intervals were also calculated. Then, classical statistical analyses were also performed to evaluate the possible association between the genetic variants of *CHRNA3* and *CHRNA5* and cigarette consumption intensity. For this analysis, we considered the total sample of individuals (cases and controls) who answered that they are former smoker and current smoker, independently of the tumor present. We adjusted the analysis for the gender, age, and cancer present. All analyses were performed using SPSS (Statistical Package for Social Sciences, v.18.0).

## 3 RESULTS

We conducted the analysis of our patients by first separating the individuals in the HNC patients and the control group. Demographic characteristics of cases and controls are summarized in Table 1, where significant differences were found between the groups for gender distribution (*p* = .004), skin color distribution (*p* = .002), age (*p* = .004), educational level (*p* = .008), and tobacco use (*p* = .001).

**Table 1:** Sociodemographic data, educational level, skin color, and tobacco consumption of head and neck cancer (619) and control (448) patients

Factor	Category	Control		Patient		χ^2^	*p*	OR	*p*	95% IC	
		*N*	%	*N*	%						
Gender	Female	71	15.8	61	9.9	8.55	.003*	1.00	—	—	—
	Male	377	84.2	557	90.1			1.72	.004*	1.19	2.48
	Missing	0	—	1	0.2						
Skin color	White	258	58.4	417	67.7	9.69	.002*	1.00	—	—	—
	No white	184	41.6	199	32.3			0.67	.002*	0.52	0.86
	Missing	6	1.3	3	0.5						
Age (years)	≤50	184	41.2	201	32.5	8.39	.004*	1.00	—	—	—
	>50	263	58.8	417	67.5			1.45	.004*	1.13	1.87
	Missing	1	0.2	1	0.2						
Education level	>11 years	60	14.1	54	8.7	7.42	.025*	1.00	—	—	—
	8–11 years	104	24.4	159	25.7			1.70	.02	1.10	2.64
	0–8	262	61.5	405	65.5			1.72	.009	1.16	2.56
	Missing	22	5.0	1	0.2						
Tobacco user	Never	119	26.7	31	5.0	134.16	<.001*	1.00	—	—	—
	Former	124	27.8	112	18.2			3.48	<.001*	2.18	5.51
	Current	202	45.4	472	76.8			8.97	<.001*	5.90	13.67
	Missing	3	0.7	4	0.6						

Abbreviation: OR, odds ratio.

* *p* < .05.

The genes studies were in Hardy–Weinberg equilibrium (data not shown). We evaluated the possible association between the *CHRNA*3 (rs578776) and *CHRNA5* (rs16969968) polymorphisms and HNC risk. However, our results did not show a statistically significant association between both SNPs and neoplasm risk, as described in Table 2.

**Table 2:** Distribution of the *CHRNA*3 (rs578776) and *CHRNA5* (rs16969968) polymorphisms of the head and neck cancer (619) and control (448) groups

		Controls		Cases		χ^2^	*p*	OR	*p*	IC 95%
		*N*	%	*N*	%					
*CHRNA3*										
Genotype										
CC		167	37.4	227	36.8	1.144	.56	1.00	—	—
CT		207	46.4	303	49.1			0.239	1.196	0.888–1.611
TT		72	16.1	87	14.1			0.746	1.071	0.706–1.625
Missing		2	0.4	2	0.3					
Allele										
C		270.5	60.6	378.5	61.35			1.00	—	—
T		175.5	39.4	238.5	38.65			0.97	.85	0.75–1.25
*CHRNA5*										
Genotype										
GG	246		55	320	51.8	1.298	.52	1.00	—	—
GA	160		35.8	242	39.2			0.54	1.096	0.818–1.469
AA	41		9.2	56	9.1			0.27	.763	0.473–1.233
Missing	1		0.2	1	0.2					
Allele										
G	326		72.9	441	71.4			1.00	—	—
A	121		27.1	177	28.6			1.08	.58	0.82–1.42

Abbreviation: OR, odds ratio.

Then, we evaluated the possible association between the polymorphisms and the cigarette consumption intensity in the total sample of individuals who answered that they are former smoker (236) and current smoker (674), independently of the tumor present. For the analysis of cigarette consumption, the sample was separated into two groups: people who smoked until 20 cigarettes per day (CPD) and people who smoked 20 or more CPD, regardless of whether they had neoplasms. The individuals who declared that they never smoked were excluded from this analysis.

The genotype frequencies of the *CHRNA3* polymorphism (rs578776) were similar in both groups. These results showed no association between the *CHRNA3* polymorphism and tobacco consumption intensity (Table 3).

**Table 3:** Relation between the *CHRNA3* (rs578776) polymorphism and tobacco consumption intensity

	Until 20 CPD	≥20 CPD	OR	*p*	IC 95%
*CHRNA3*					
Genotype					
CC	76 (33.8%)	261 (38.7%)	1.00	—	—
CT	112 (49.8%)	323 (47.8%)	0.84	.31	0.60–1.17
TT	37 (16.4%)	91 (13.5%)	0.72	.18	0.45–1.14
Allele					
C	132 (58.7%)	422.5 (62.6%)	1.00	—	—
T	93 (41.3%)	252.5 (37.4%)	0.85	.30	0.63–1.16

Abbreviations: CPD, cigarettes per day; OR, odds ratio.

In relation to the *CHRNA5* (rs16969968) SNP, our results showed that the homozygous genotype was associated with increased cigarette consumption intensity (OR 1.93, 95% CI 1.05–3.58), as shown in Table 4.

**Table 4:** Relation between *CHRNA5* (rs16969968) polymorphism and tobacco consumption intensity

	Until 20 CPD	≥20 CPD	OR	*p*	IC 95%
*CHRNA5*					
Genotype					
GG	118 (52.4%)	348 (51.4%)	1.00	—	—
GA	94 (41.7%)	254 (37.6%)	0.91	.63	0.67–1.25
AA	13 (5.9%)	74 (11%)	1.93	.03	1.05–3.58
Allele					
G	165 (73.25%)	475 (70.2%)	1.00	—	—
A	60 (26.75%)	201 (29.8%)	1.16	.39	0.82–1.65

Abbreviations: CPD, cigarettes per day; OR, odds ratio.

## 4 DISCUSSION

Cigarette smoking has been considered the main risk factor for HNC because cigarettes have many substances with carcinogenic potential (Colombo & Rahal, 2009; Simard et al., 2014). Nicotine is the major substance present in the cigarette and binds directly to nAchRs. Several studies have shown associations between polymorphisms present in genes that encode the subunits of these receptors, such as *CHRNA3* (rs578776) and *CHRNA5* (rs16969968), and cigarette consumption and/or HNC risk (Anantharaman et al., 2014; Chen et al., 2011; Doyle et al., 2014; Jensen et al., 2015; Lips et al., 2010; Munafò et al., 2012; Qu et al., 2016; Wen et al., 2014).

In the present study, we did not find any association between the polymorphisms of the *CHRNA3* (rs578776) and HNC risk or with the quantity of cigarette consumed. The variant of *CHRNA3* (C > T) is present in the 3’ untranslated region of the gene and contains regulatory sequences (Bierut et al., 2008; Conlon & Bewick, 2011). Whether this polymorphism results in a functional change or if this tag is another polymorphism in the same region is not clear (Conlon & Bewick, 2011). Some studies have suggested that the C allele is considered a risk allele because the presence of an increased activation circuit that may be sensitive to nicotine exposure and the presence of the T allele may have a protective effect because of normal rewarding sensibility, that is, in relation to the wild allele, the polymorphic allele eases the reward system (Robinson et al., 2013). However, functional studies are necessary to elucidate the real function of this variant.

In relation to the rs16969968 polymorphism, we did not find an association between the variant genotypes and the risk of HNC, but we found an association between the mutated homozygous genotype and increased cigarette consumption (OR 1.93, 95% CI 1.05–3.58), suggesting that the variation in *CHRNA5* acts indirectly in the risk of HNC. The *CHRNA5* (G > A) polymorphism results in an amino acid change from aspartic acid to asparagine in the nicotine acetylcholine subunit α5. Studies suggest that the presence of asparagine confers a decreased interaction of the receptor with nicotine and our agonists than the presence of wild subunit α5, with reduced channel permeability and faster desensitization (Bierut et al., 2008; Stephens et al., 2013). The association of the polymorphism and increased cigarette consumption has also been found in other populations, including European American, African American, Central European, Latin Americans, and Romans (Jensen et al., 2015; Lips et al., 2010). Some studies suggest that the mutated allele is related to a decrease in the stimulatory effects of nicotine, and thus, people with this allele would consume more of the substance to have the same effects (Wen et al., 2014). Another study posits that the allele diminishes the aversive effects of nicotine and that people can consume more of the substance (Fowler & Kenny, 2014; Jensen et al., 2015). Once the α5 subunit is abundantly expressed in dopaminergic neurons (Changeux, 2010) and it modulates dopamine release, individuals with the mutated subunit α5, which have the lowest affinity for nicotine, may require higher nicotine consumption to achieve the same pathways for dopamine release (Bierut et al., 2008; Van Skike et al., 2016).

In conclusion, the *CHRNA3* (rs578776) variant was not associated with HNC risk or with the cigarette consumption intensity in our sample population. In these Brazilian samples, the polymorphism *CHRNA5* (rs16969968) was associated with increased intensity of cigarette smoking, acting as an indirect factor for HNC risk.

## CONFLICT OF INTEREST

None declared.
